# Case report: the etiology of anterior inferior cerebellar artery infarction: what does basi-parallel anatomic scanning magnetic resonance imaging tell us?

**DOI:** 10.1186/s12883-021-02309-2

**Published:** 2021-07-30

**Authors:** Zhi-yong Zhang, Zhi Zhou, Hai-bo Zhang, Jin-song Jiao

**Affiliations:** 1 Department of Neurology, Beijing Geriatric Hospital, 118 Wenquan Road, Haidian District, Beijing, 100095 China; 2 Department of Neurology, China-Japan Friendship Hospital, Beijing, 100029 China; 3 Department of Radiology, China-Japan Friendship Hospital, Beijing, 100029 China

**Keywords:** Etiology, Anterior inferior cerebellar artery, Stroke, Vertebrobasilar artery, Basi-parallel anatomic scanning magnetic resonance imaging

## Abstract

**Background:**

The precise etiology of anterior inferior cerebellar artery (AICA) infarction is difficult to identify because of the high anatomic variability of vertebrobasilar arteries and the limitations of conventional vascular examinations. Basi-parallel anatomic scanning magnetic resonance imaging (BPAS-MRI) can reveal the outer contour of the intracranial vertebrobasilar arteries, which may be helpful to distinguish the arteriosclerosis from congenital dysplasia and dissection.

**Case presentation:**

In this study, we reported 3 cases of AICA infarction and discussed the diagnostic value of BPAS-MRI in the evaluation of vascular etiology.

**Conclusions:**

The BPAS-MRI could be considered as an important supplementary in the diagnosis of vascular etiology of infarction in AICA territory.

## Background

The anterior inferior cerebellar artery (AICA) infarction may cause vertigo, hearing loss, facial weakness, Horner’s syndrome, ataxia or other neurological symptoms. As the treatments might vary due to different etiologies, the accurate identification of etiology is of great significance to the management. Conventional vascular imaging methods, including the time-of-flight magnetic resonance angiography (TOF-MRA) and computed tomographic angiography (CTA), have limitations in the diagnosis of AICA's own lesions. First, AICA is not typically visualized on CTA due to the limited detection of the smaller branches [1]. Second, as these methods can only show the narrowing or absence of an artery with the illustration of inner vascular wall, it’s difficult to distinguish the stenosis or occlusion from hypoplasia or aplasia because of the high anatomic variability in vertebrobasilar artery system [2]. Therefore, due to the uncertainty of AICA displayed by routine vascular examination, the etiology of AICA infarction is more confusing. Basi-parallel anatomic scanning magnetic resonance imaging (BPAS-MRI) can reveal the outer contour of the vertebrobasilar arteries and has good value for differential diagnosis in atherosclerotic stenosis, hypoplasia and dissection, which may be of help for the diagnosis of AICA lesions [3, 4]. We herein present 3 cases with AICA infarction and discuss the value of BPAS-MRI in the diagnosis of vascular etiology of AICA infarction.

## Case presentation

### Case 1

**Fig. 1:**
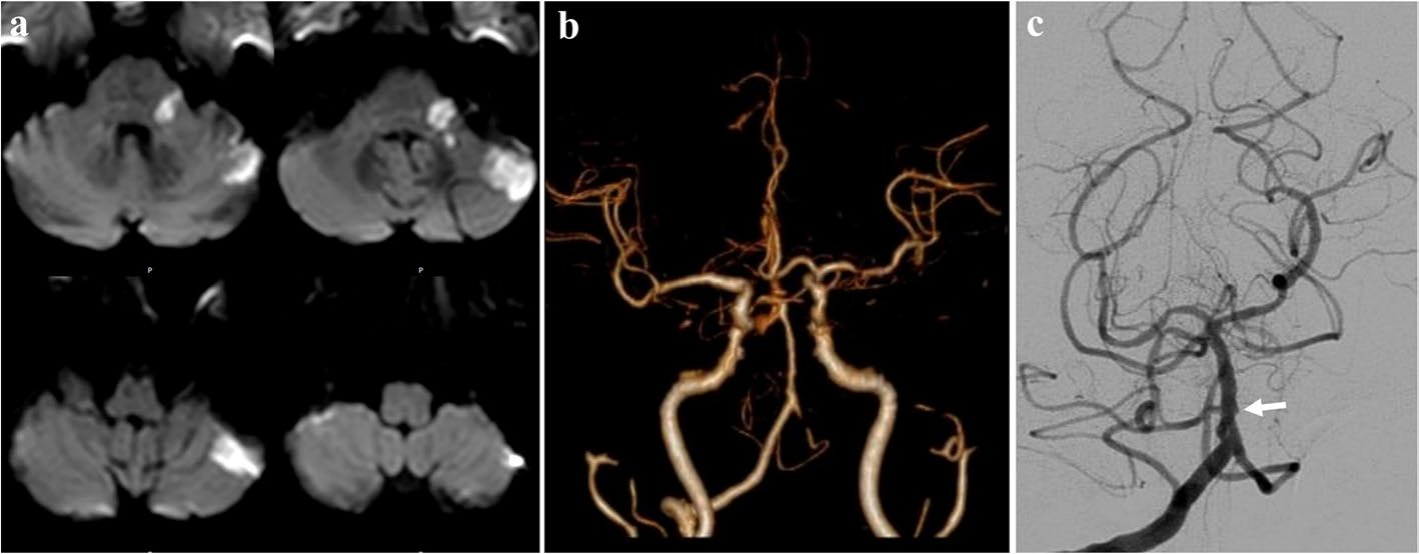
Diffusion-weighted image revealed acute infarction on left brachium pontis and left superior lateral cerebellum (**a**). Computed tomographic angiography showed the server stenosis and occlusion of left veterbral artery and the absence of bilateral anterior inferior cerebellar artery (AICA) (**b**). Digital subtraction angiography showed occlusion from left proximal AICA (**c**)

A 69-year-old woman with history of diabetes mellitus and hypertension presented with sudden vertigo, left hearing loss, and left ataxia. MRI showed hyperintensity in left brachium pontis and left superior lateral cerebellum on diffusion-weighted image (DWI) (Fig. 1a), indicating an acute infarction in left AICA territory. CTA showed the server stenosis and occlusion of left veterbral artery and absence of bilateral AICA (Fig. 1b). Digital subtraction angiography (DSA) showed occlusion from left proximal AICA (Fig. 1c). Combined with the history, the etiology was presumed as atherosclerotic occlusion of left AICA.

### Case 2

**Fig. 2:**
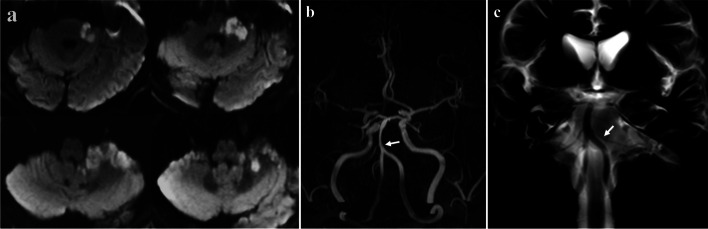
Diffusion-weighted image revealed left anterior inferior cerebellar artery (AICA) territorial infarction (**a**).On magnetic resonance angiography, the left AICA was absent, while the right AICA was visualized clearly (**b**). Basi-parallel anatomic scanning magnetic resonance imaging (BPAS MRI) showed normal bilateral AICA (**c**)

A 64-year-old male patient with hypertension presented with vertigo, left peripheral facial palsy and left ataxia. DWI revealed left AICA territorial infarction (left inferolateral pons, brachium pontis, superior cerebellum) (Fig. 2a). On MRA, the left AICA was absent, while the right AICA was visualized clearly (Fig. 2b). BPAS-MRI showed normal bilateral AICA (Fig. 2c). Therefore, the radiological diagnosis of the left AICA occlusion was made, and left AICA was the victim vessel of this infarction.

### Case 3

**Fig. 3:**
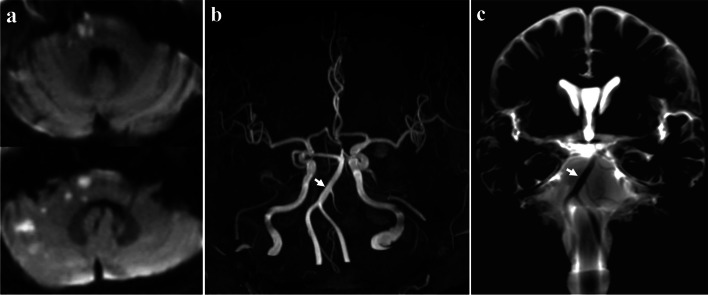
The diffusion-weighted image showed hyperintensity lesions in right inferolateral pons, brachium pontis and superior cerebellum (**a**). The right anterior inferior cerebellar artery (AICA) was absent on magnetic resonance angiography (**b**) and basi-parallel anatomic scanning magnetic resonance imaging (BPAS-MRI) (**c**)

A 60-year-old male patient presented with vertigo and right ataxia. The DWI showed acute infarction involving right inferolateral pons, brachium pontis and superior cerebellum (Fig. 3a). The right AICA was absent on MRA (Fig. 3b) and BPAS-MRI (Fig. 3c). The comprehensive diagnosis was considered as right AICA congenital aplasia, and other causes of the infarction needed to be explored. Finally, a proximal atrial fibrillation was found through long-term monitoring, and the cardiac embolism was presumed as the etiology of infarction.

## Discussion

The etiology of AICA infarction is difficult to diagnose due to the high anatomic variability in vertebrobasilar system, even both of the patients in Case 1 and Case 2 had atherosclerotic risk factors in our study. It’s reported that only 35% of patients had normal vestebrobasilar arteries, and approximately 29% of the posterior circulation anatomic variability is related to AICA [5]. In Case 1, if the occlusion was from the origin of AICA instead of the proximal part, even the DSA, which has widely been considered as the gold standard, still could not differentiate the occlusion from congenital dysplasia.

Compared with conventional methods, such as TOF-MRA, CTA or DSA, BPAS-MRI could provide an accurately visualization of the outer contour of vascular wall regardless the influence of blood flow or thrombus, even when the artery is occluded. In Case 2, the absent of left AICA on TOF-MRA and the presence of AICA on BPAS-MRI imaging led to a straightforward diagnosis of AICA occlusion due to atherosclerosis or thrombus. This suggested the supplementary role of BPAS-MRI to TOF-MRA in the diagnosis of AICA occlusion. In Case 3, though the topography of infarction was consistent with AICA territory, the absence of AICA on MRA and BPAS-MRI indicated the congenital aplasia of AICA. If we simply attributed the cause to AICA occlusion, we might miss the real etiology and take inappropriate treatment.

In the future, large-scale case studies with multiple imaging modalities are needed to validate the supplementary role of BPAS-MRI in the diagnosis of etiology of AICA.

## Conclusions

The BPAS-MRI could be considered as an important supplementary in the diagnosis of vascular etiology of infarction in AICA territory.

## Data Availability

The data used during the current study are available from the corresponding author on reasonable request.

## Abbreviations

AICA: Anterior inferior cerebellar artery
TOF-MRA: Time-of-flight magnetic resonance angiography
CTA: Computed tomographic angiography
BPAS-MRI: Basi-parallel anatomic scanning magnetic resonance imaging
DWI: Diffusion-weighted image
DSA: Digital subtraction angiography
